# A Multifractal-Guided Machine Learning Framework for Late Post-Traumatic Seizure Prediction Following Hemorrhagic Traumatic Brain Injury

**DOI:** 10.21203/rs.3.rs-8613721/v1

**Published:** 2026-01-19

**Authors:** Daria Riabukhina, Kseniia Kriukova, Paul M. Vespa, Manuel B. Blanco, Paul Bogdan, Dominique Duncan, Emily A. Pereira

**Affiliations:** 1 Ming Hsieh Electrical and Computer Engineering Department, University of Southern California, Los Angeles, CA 90031, USA.; 2 Department of Neurology, Perelman School of Medicine, University of Pennsylvania, Philadelphia, PA 19104, USA.; 3 Brain Research Institute, University of California Los Angeles, Los Angeles, CA 90095, USA.; 4 Department of Neurosurgery, David Geffen School of Medicine, University of California Los Angeles, Los Angeles, CA 90095, USA; 5 Department of Electrical and Computer Engineering, Texas Tech University, Lubbock, TX 79409, USA.

## Abstract

Traumatic brain injury can lead to post-traumatic epilepsy, yet early, reliable biomarkers to predict its emergence remain elusive. By investigating the multifractal characteristics of electroencephalogramrecordings from the first available day post-injury, we develop for the first time a machine learning framework that distinguishes between traumatic brain injury patients who develop late post-traumatic seizures and those who do not. Statistical analysis demonstrates statistically significant differences in multifractal properties of EEG signals between patients who develop late post-traumatic seizures and patients who do not. We show that random forest classifier trained on multi-fractal properties of EEG achieve a high predictive accuracy (95%) and area under the curve (98%) for predicting late PTS. The predictive power of multifractal features was robust to sample length and electrode selection. Our findings indicate that multifractal properties of EEG offers a promising, objective approach to early risk stratification for post-traumatic epilepsy in neurocritical care settings.

## Introduction

Traumatic brain injury (TBI) is among the most common and serious injuries, representing a major global public health concern; its incidence worldwide is estimated at 69 million cases annualy^1–5^. Post-traumatic epilepsy (PTE) is a major long-term complication of TBI, representing one of the leading causes of acquired epilepsy worldwide. The risk of developing PTE is strongly associated with the severity and type of TBI. According to recent research, the incidence of PTE following severe TBI can be substantial, with some studies reporting rates as high as 30–50%, especially in cases involving penetrating injuries or extensive cortical damage^6–8^. PTE is defined by the onset of unprovoked seizures occurring more than seven days after TBI, known as late post-traumatic seizures (PTS). In contrast, seizures within 24 hours (immediate PTS) or within seven days (early PTS) are considered provoked and do not meet criteria for epilepsy^9–11^. In severe TBI, a single late PTS is sufficient for a diagnosis of epilepsy due to the high recurrence risk^8, 12–14^. The onset of PTE remains highly unpredictable - some patients may develop epilepsy within days of injury, while in others, seizures may not emerge until months or even years later^15–17^.

Electroencephalography (EEG) is a widely accessible tool that shows promise for predicting PTE and studying brain activity in TBI survivors^18–21^. Although significant efforts have been made to identify predictive EEG biomarkers, none have been validated for clinical use to date^20, 21^.

EEG signals exhibit a high degree of complexity marked by strong non-stationarity, non-Markovianity and multifractal patterns^22^. Their observed nonstationary and fractal properties mean that the standard linear methods cannot capture their full complexity^23–25^. The underlying neuronal activation generating EEG is likely nonlinear, and thus, traditional methods of EEG analysis may miss many properties inherent in such signals.^26^. The brain’s electrical signals contain subtle patterns (e.g. bursts, oscillation amplitude modulation, scale-free fluctuations) that span multiple time scales, challenging us to go beyond standard single-scale descriptors. While standard measures like power spectral density can assess the distribution of EEG activity across frequency bands (e.g., delta, theta, alpha, beta), they primarily assume stationarity and capture only second-order statistics^27, 28^. Moreover, this approach can miss dynamic or nonlinear features of the EEG signal^29–33^. For example, Hardstone et al. (2012) demonstrated that amplitude dynamics specifically, long-range correlations are largely independent of mean oscillation power, suggesting that scale-free metrics provide additional, unique information beyond what PSD reveals^27, 29^. Crucially, brain dynamics are inherently non-linear and rank among the most complex natural phenomena, spanning a wide range of interacting time scales from fast electrical oscillations to slower chemical and diffusive processes^34, 35^.

Studies indicate that such neural processes often display “scale-free” characteristics, where certain features of the signal exhibit self-similarity across multiple time scales^36, 37^. Fractal geometry provides a useful framework to describe this scale invariance, with a single fractal exponent (or fractal dimension) capturing the properties of self-similar, or “monofractal,” signals^33, 38^. However, physiological and real-world signals, including EEG, typically demonstrate more complex, multifractal behavior, with different subsets of fluctuations characterized by their own distinct fractal dimensions^39, 40^. Thus, multifractal analysis has proven to be helpful in capturing these diverse dynamics and has shown promise in differentiating brain activity in both health and disease, including epilepsy^34, 41^.

**Figure 1.**
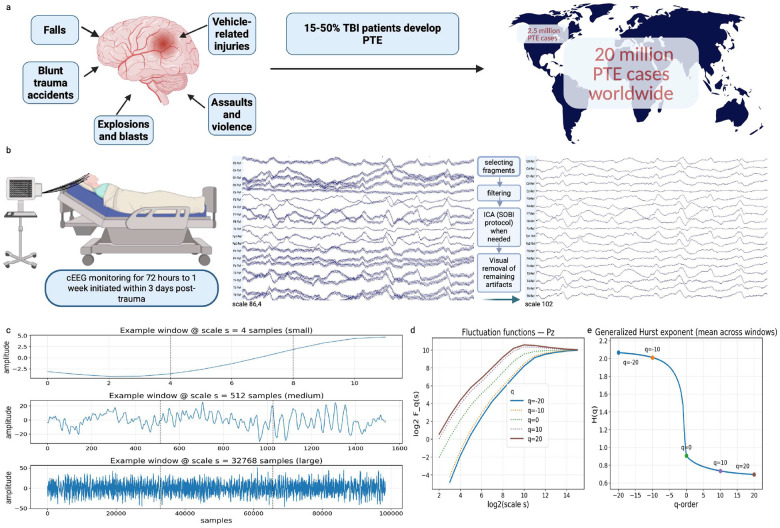
Overview of the proposed method for seizures prediction: a) PTE epidemiology b) EEG data acquisition and EEG data preprocessing pipeline c) examples of non-overlapping segments partitioning the signal profile d) for each scale the fluctuation function is computed e) generalized Hurst exponents, determined from the scaling behavior of the fluctuation function, from the first available day, using a sliding window and finding day average values

Multifractal detrended fluctuation analysis (MFDFA) is commonly used to evaluate fractal properties in EEG signals and can detect subtle changes in neuronal dynamics^29, 40, 42^. Traditionally, such analyses have focused on predicting imminent seizures, detecting changes in EEG minutes before seizure onset^43^. In moderate to severe TBI, normal brain rhythms are often disturbed by factors such as intracranial hemorrhage, brain contusion, edema, along with altered consciousness, and medications, potentially producing unique fractal signatures that might signal early epileptogenic processes. We hypothesize that disruptions in fractal scaling or multifractal spectra may serve as early markers of neuronal network dysfunction and could be linked to an increased risk of PTE in TBI patients. In this work, we quantify mulit-fractal properties of EEG data using MFDFA from a unique TBI patient cohort, where the timing of epilepsy development may occur days to years after injury, which to our knowledge, represents the first attempt to characterize multi-fractal properties in TBI patients. Furthermore, we develop a machine learning framework to classify TBI patients who develop late PTS by leveraging multi-fractal properties of EEG data. Figure 1 summarizes the process we used to predict late PTS. By systematically analyzing these features in EEG data, we show a novel biomarker to predict late PTS that may enhance prognostic accuracy and support timely clinical intervention.

TBI initiates a cascade of multiscale biological processes spanning molecular signaling, cellular excitability, network reorganization, and large-scale brain dynamics. From a complex systems perspective, epileptogenesis following TBI can be viewed not as the consequence of a single lesion or discrete abnormality, but as the emergence of a pathological dynamical regime arising from disrupted interactions across spatial and temporal scales. EEG activity provides a macroscopic window into these dynamics, capturing the collective behavior of neuronal populations whose interactions exhibit strong nonlinearity, long-range temporal correlations, and scale-free organization. While prior EEG-based approaches have largely focused on spectral or morphological features, such methods primarily reflect second-order statistics and may fail to capture higher-order temporal dependencies characteristic of complex adaptive systems. In this study, we hypothesize that early post-injury alterations in the multiscale organization of brain activity, quantified through multifractal scaling properties of EEG, reflect an underlying loss of dynamical stability that predisposes certain patients to the later emergence of seizures. By integrating multifractal analysis with machine learning, we aim not only to improve early prediction of late post-traumatic seizures, but also to provide insight into how acute disruptions of multiscale brain dynamics may signal long-term pathological reorganization.

## Methods

### EpiBioS4Rx EEG Dataset

We studied EEG data collected from the EpiBioS4Rx study - a multicenter, longitudinal epilepsy bioinformatics study aimed at developing anti-epileptogenic therapies^44^. Patients included in the dataset experienced an acute TBI with evidence of intracranial, cortical, and/or subcortical bleeding on computed tomography (CT) imaging. Participants ranged in age from 6–100 and had Glasgow Coma Scale (GCS) scores of 3–13 without continuous sedation.

**Figure 2.**
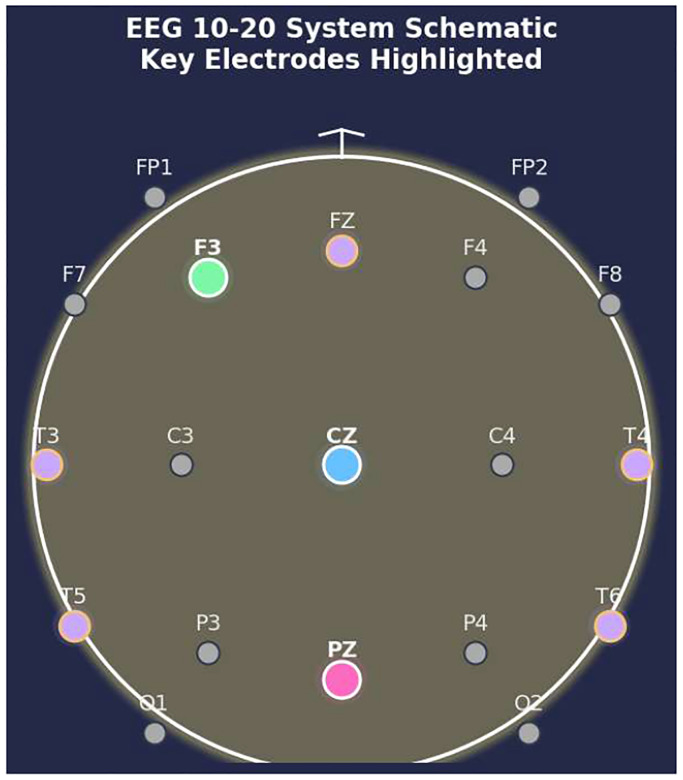
Locations of electrodes used to collect the data

At the time of initiating this analysis, outcomes were available for 103 patients. 31 patients were excluded due to poor data quality, and six patients were excluded due to missing data. Of the remaining patients, 33 developed late PTS, while 33 did not. Table 1 gives a detailed description of the TBI patient data. Continuous EEG (cEEG), including surface and, in some cases, intracranial monitoring, was performed as a standard of care at each site. Upon admission and during consideration for enrollment in the EpiBioS4Rx study, cEEG monitoring adhered to standard practices, lasting from 72 hours up to one week. Both cap and needle electrodes were utilized, and a referential montage was applied. The number of electrodes varied; the minimum was 12, and they were placed according to the 10–20 system, shown in Figure 2.

Initial visual cEEG analysis was performed using EDFbrowser. The data were initially filtered with high-pass filter frequency at 1 Hz low-pass filter at 250 Hz and 500 Hz, and a notch filter at 60 Hz and its harmonics (FIR filter) using Fieldtrip functions^45^. The data were recorded using referential montage. Bad channels with poor signal quality as well as channels representing intracranial EEG (iEEG) were identified and removed, as iEEG was not considered for the current analysis. Using the MATLAB-based toolbox EEGLAB, Independent Component Analysis with the Second-order Blind Identification protocol was applied to the EEG data to remove artificial components when necessary. Manual artifact rejection was applied to the EEG data by a trained neurologist to remove non-physiological fragments^46^.

### Multifractal Detrended Fluctuation Analysis

We use multi-fractal detrended fluctuation analysis (MFDFA)^42^ to characterize the fractal properties of EEG signals for each patient. Let $x_{k}$ be a signal from one EEG channel. We construct

(1) $$Y\left(i\right)=\sum_{k=1}^{i}\left(x_{k}-\overset{‾}{x}\right),\;i=1,\ldots ,N.$$

where $\overset{‾}{x}$ is the mean for $x_{k}$ and N is its length. For a given scale $s\in \left\{2^{4},2^{5},2^{6},\ldots \right\},Y(i)$ is partitioned into $N_{s}=\lfloor N/s\rfloor$ non-overlapping segments. In each segment $v=1,\ldots ,N_{s}$ a polynomial trend $y_{v}(i)$ is removed. The local variance is

(2) $$F^{2}\left(s,v\right)=\frac{1}{s}\sum_{i=1}^{s}{\left[Y\left(\left(v-1\right)s+i\right)-y_{v}\left(i\right)\right]}^{2}.$$

The fluctuation function is defined for q≠0:

(3) $$F_{q}(s)={\left\{\frac{1}{N_{s}}\sum_{v=1}^{N_{s}}{\left[F^{2}(s,v)\right]}^{q/2}\right\}}^{1/q},\;q\neq 0,$$

and at q=0:

(4) $$F_{0}(s)=\text{exp}\left\{\frac{1}{2N_{s}}\sum_{v=1}^{N_{s}}\text{ln}\left[F^{2}(s,v)\right]\right\}.$$

We consider

(5) $$F_{q}(s)\propto s^{h(q)},$$

and the generalized Hurst exponent h(q) is obtained as the slope of the linear regression^40, 42^.

(6) $$h(q)=\frac{d\;\text{log}\;F_{q}(s)}{d\;\text{log}\;s}$$

For each channel, we computed generalized Hurst exponents (h(q)) inside non-overlapping sliding windows of 100,000 samples. Within each window, we used the scale $s\in \left\{2^{2},2^{3},\ldots ,2^{15}\right\}$, and computed $F_{q}(s)$ for q∈{−20,−19.5,…,19.5,20}, which totals 81 values of q. Hence, we computed 81×W matrix of generalized Hurst exponents corresponding to 81 values of q from −20 to 20 with a stepsize 0.5 and W is the number of 100,000-sample windowsfor each patient. Hence, W is variable for each patient. We averaged the 81×W matrix of generalized Hurst exponents across time windows (W) to obtain an averaged vector of generalized Hurst exponents of length 81.

We also computed generalized Hurst exponents for various lengths of the total amount of data ranging from 20 minutes to 1 hour, which showed that the results do not vary greatly when considering more data (see Supplementary Material). Complexity of the algorithm is O(N⋅S⋅Q), where N is the length of time series, S is the number of scales, and Q is the number of q-exponents.

### Kolmogorov-Smirnov Test with False Discovery Rate Correction

To address multiple comparisons in our mass-univariate EEG analyses, we controlled the false discovery rate (FDR) at q=0.05 using the Benjamini–Hochberg procedure.

### Classification Pipeline

Since we have different sets of channels for each patient, we treat every channel as an independent sample. For each patient, we analyzed EEG data only from the first day available, otherwise the data may be highly correlated. The average amount of channels per patient is 14.71 ± 3.10. Our features include the averaged (across time windows) generalized Hurst exponents computed from EEG data from the first available day for q from −20 to 20 with a stepsize 0.5 as well as age and Glasgow Coma Scale (GCS) score from the first available day. We trained and tested four classifiers, including logistic regression, support vector machine (SVM), random forest, and a deep neural network (DNN). For the random forest classifier, we applied Principal Component Analysis (PCA) to the features and trained it on the first 5 PCA components. We trained the random forest model using 100 trees and the Gini impurity splitting criterion. The class weight was set as balanced. Fully-connected DNN has three hidden layers with ReLU activation functionsand 30 % dropout after each layer; the fourth hidden layer has a sigmoid activation function. We used 5-fold stratified cross-validation with shuffled data to test our classifiers. Hyperparameter tuning was performed for all the models, but for SVM we made a very precise grid search as this model depends heavily on hyperparameters choice.

### Baseline Model

We used the method from^19^ as a baseline model to compare with our results. We analyzed EEG data from the first available day, using all the channels available. We downsampled the data to 250 Hz. The EEG signal x[t] was segmented into 2-second Hann windows $w[n]=0.5-0.5cos\left(\frac{2\pi n}{N_{\text{seg}}-1}\right)$, where $N_{\text{seg}}=\left\lfloor 2\cdot f_{s}\right\rfloor$ for sample frequencies $f_{s}=250\;\text{Hz},n$ ranges from 0 to $N_{\text{seg}}-1$, with a 50% overlap. T=2 seconds was chosen, since $\Delta f=\frac{1}{T}=0.5\;\text{Hz}$ allows us to have several Power Spectral Density (PSD) bins in the shortest frequency δ bands of 1–4 Hz. We computed PSD using Welch’s method

(7) $$P_{xx}(f)=\frac{1}{K}\sum_{k=1}^{K}\frac{{\left|ℱ\left[x_{k}[n]w[n]\right]\right|}^{2}}{U},$$

where K is the number of windows, $U=\frac{1}{N_{\text{seg}}}\sum_{n}w^{2}[n]$ is the window normalization factor, ℱ{⋅} is the Fourier transform. We computed absolute bandpower

(8) $$BP_{B}=\int_{f_{1}}^{f_{2}}P_{xx}(f)df$$

for each EEG band $B=\left(f_{1},f_{2}\right):\delta (1-4\;\text{Hz}),\theta (4-8\;\text{Hz})$. Finally, we found the amplitude envelope

(9) $$|a(t)|=\sqrt{x^{2}(t)+{ℋ}^{2}[x(t)]},$$

where ℋ{⋅} is Hilbert transform, which was included because there is evidence that the amplitude envelope significantly improves performance in similar cases^47^. The features for the baseline model that we used are listed in Table 2.

We compared four classifiers for predicting late PTS, including logistic regression, support vector machine (SVM) with radial basis function and grid search, and deep neural network (DNN).

## Results

### Increased Multifractality Evident in EEG Data from patients with Late PTS

**Figure 3.**
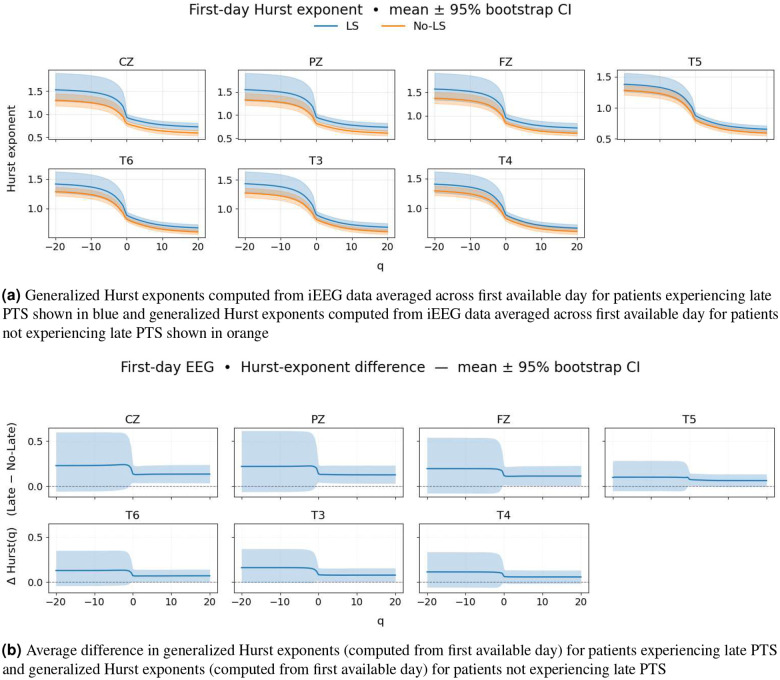
Generalized Hurst exponents (computed from first available day) for patients experiencing late PTS and patients not experiencing late PTS

Figure 3a shows the generalized Hurst exponents computed from EEG data for patients who experience late PTS (blue) and patients who do not experience late PTS (orange). The transparent areas represent the bootstrap confidence interval 95%. The generalized Hurst exponents for patients with late PTS are not significantly separable from generalized Hurst exponents for patients without late PTS; however, for many electrodes we observe a difference for small and large q values, indicating differences in multifractal properties (see Figure 3). The difference in generalized Hurst exponents at small q values and large q values (i.e., $H\left(q_{\text{min}}\right)-H\left(q_{\text{max}}\right)$) tends to be larger for patients experiencing late PTS, indicating stronger heterogeneity in fractal properties across time. Bootstrap bands show higher variability for patients experiencing late PTS for q=20 and q=−20. Some channels, such as T3, T4, T5, and T6, do not demonstrate a large difference between generalized Hurst exponents for patients experiencing late PTS as compared with generalized Hurst exponents for patients who do not experience late PTS (see Figure 3). On the other hand, channels such as CZ, PZ, FZ, do exhibit a large difference between generalized Hurst exponents for patients experiencing late PTS as compared with generalized Hurst exponents for patients who do not experience late PTS (see Figure 3).

Figure 3b shows the difference between the population means of the generalized Hurst exponents for patients who experience late PTS and the population means of the generalized Hurst exponents for patients who did not experience late PTS for seven most significant channels. The average difference between the generalized Hurst exponents ($\Delta H(q)={\overset{-}{H}}_{\text{Seizures}}(q)-{\overset{-}{H}}_{\text{NoSeizures}}(q)$) is shown as a solid line, and the bootstrap confidence interval built with 4000 samples is a translucent area. A gray dashed line marks where there is no difference between generalized Hurst exponents (ΔH=0). The differences are positive for the majority of q values, which indicates that EEG data collected from patients experiencing late PTS can be characterized with greater multi-fractality. We observe a non-smooth difference between generalized Hurst exponents at q=0 due to the piecewise definition of the fluctuation function (see Equations (3) and (4)).

For most time window length selections, the generalized Hurst exponents are separable to some degree, which is demonstrated in Supplementary Material. Hence, there is nothing special about choosing a particular time window to compute the generalized Hurst exponents. For our classification results, we used all the data from the first available day, which vary in length for each patient.

### Statistical Evidence for Distinguishability between Multifractal Properties of EEG from Patients with late PTS and Patients without late PTS

**Figure 4.**
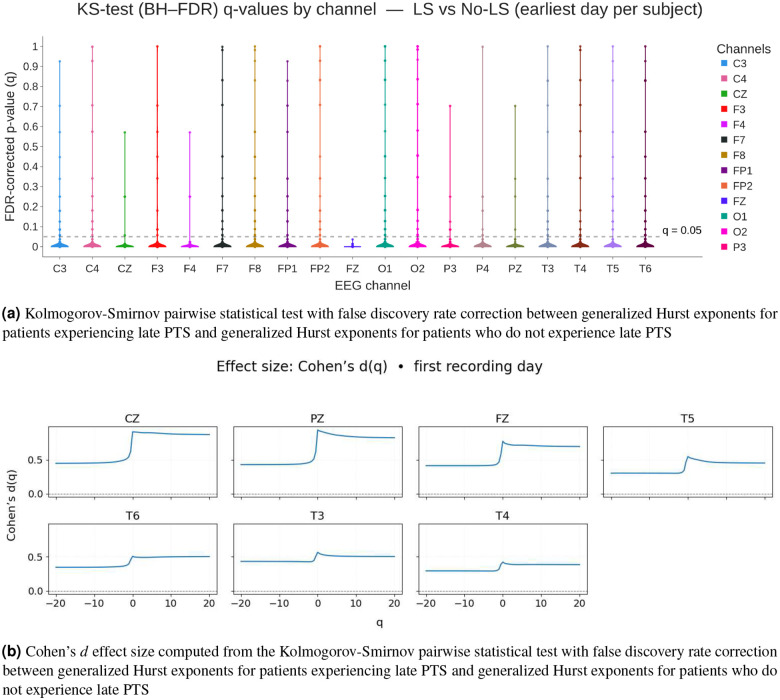
Kolmogorov-Sminnov test between generalized Hurst exponents computed during first available day of iEEG data from patients experiencing late PTS and patients who do not experience late PTS

Figure 4a shows the results of the Kolmogorov-Smirnov tests in pairs with false discovery rate (FDR) correction between generalized Hurst exponents for patients experiencing late PTS and generalized Hurst exponents for patients who do not experience late PTS. We observe that more than 95% of p-values for each channel remain significant after FDR. While there are some p-values greater than 0.05, less than 5% of p-values are not statistically significant for each electrode. The median p-values for all channels are in the interval 10^−6^ −10^−7^.

Figure 4b shows the Cohen’s effect size between generalized Hurst exponents for all q computed from EEG data from patients with late PTS and generalized Hurst exponents for all q computed from EEG data from patients wihtout late PTS. Choen’s d values mainly fall in the moderate region, which is in consistent with the results observed in Figure 3a, where the two groups are visibly separated, but not remarkably different. The non-smooth behavior at q=0 is consistent with the observations in Figure 3b. For Cohen’s d effect size, the mean difference is divided by the pooled standard deviation, which is smaller around q=0 because of the definition of the fluctuations at q=0 thereby creating the non-smooth trend. We consider these results to be very promising for classifying patients who develop late PTS and those who do not.

### Accurate Prediction of Late PTS with Multifractal EEG Features

Table 3 shows the classifiction results for four classifiers, including logistic regression, support vector machine (SVM), random forest, and deep neural network (DNN). We computed the average performance metrics in Table 3 using 5-fold stratified cross-validation, where the range indicates the standard deviation.

**Figure 5.**
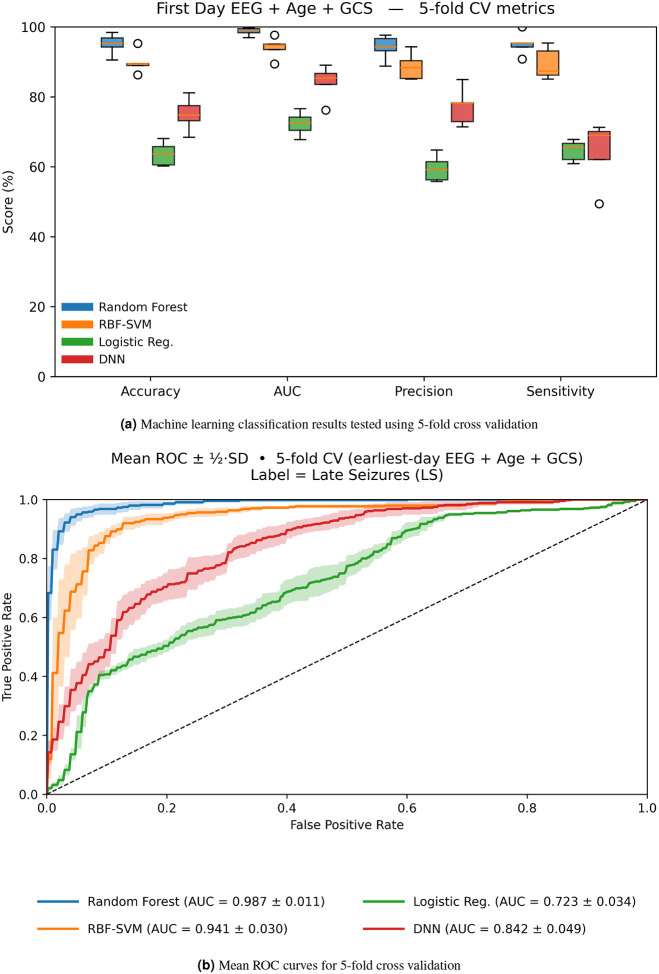
Performance results of Our model trained on generalized Hurst Exponents

Figure 5a shows the performance of each machine learning model on all 5 folds. The random forest classifier outperforms the other models with an average accuracy of 95.04% and average AUC of 98.83% with small standard deviation, indicating precise classification and low variability between folds. Random forest demonstrates high precision and sensitivity values, which shows that the model is good at identifying positive cases without generating false alarms.

The random forest model is suitable for capturing nonlinear interactions expected from generalized Hurst exponents due to its architecture. The logistic regression model reports the lowest scores and a relatively large standard deviation due to its inability to capture nonlinear relations in the data; however, it does exhibit the fastest runtime. Still, the fast runtime does not outweigh the higher classification accuracy exhibited in the random forest model. The support vector machine (SVM) with Gaussian kernel performs much better than logistic regression, but it still has a slightly worse performance than the random forest model due to its inability to capture locally correlated features. Finally, the deep neural network (DNN) does not perform much better than logistic regression model likely due to the relatively small dataset.

All of these machine learning models have relatively low runtimes, if we do not include hyperparameters grid search. The most computationally intensive step in our analysis is computing the generalized Hurst exponents, which may increase in runtime depending on the length of data and the length of non-overlapping sliding windows.

Figure 5a shows that the random forest model gives the best results with low spread, which indicates stability. SVM is close behind the performance of the random forest model, demonstrating the supremacy of nonlinear models in predicting late PTS. Figure 5b shows receiver-operating-characteristic (ROC) curves for the 5 folds. Our random forest and SVM models clearly outperform other models showing less variability across folds.

Finally, our classification results demonstrate independence from the length of the samples. In the Supplementary Material, we demonstrate that the classification performance is similar using generalized Hurst exponents computed using the first 10 minutes and the first one hour of the data available.

### Low Accuracy and Unstable Performance in a PSD-Based Model

**Figure 6.**
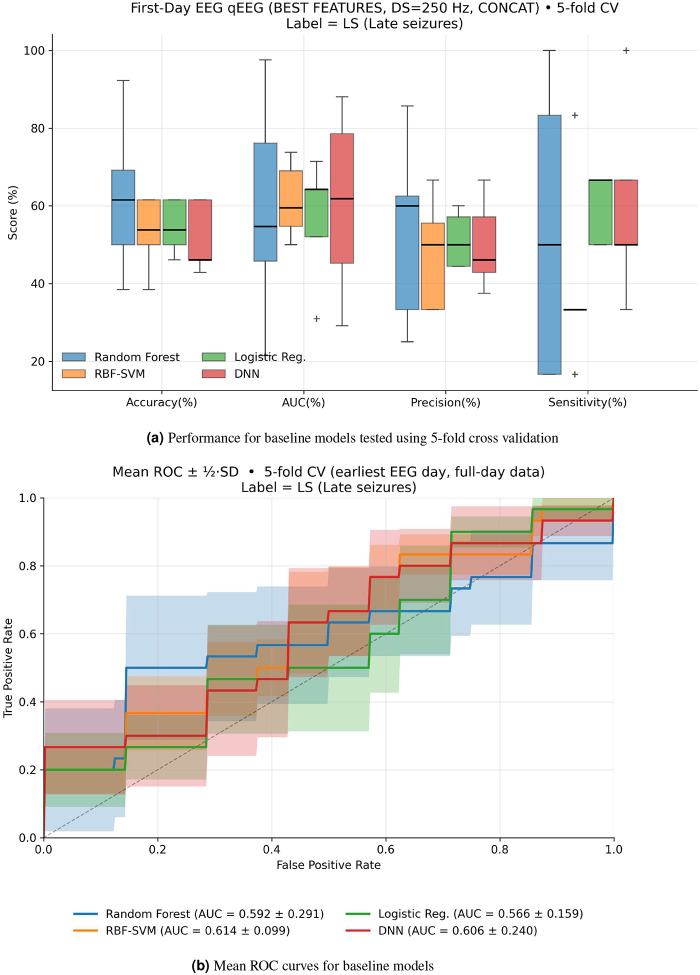
Baseline machine learning classification results

The baseline clasification model, trained on power spectral features shown in Table 2, performed significantly worse than our model (see Table 4 and Figure 6a) with the highest average accuracy of 50.88 ± 15.17 from the random forest model. In Figures 6a and Figure 6b, the baseline models trained with power spectral density features showed very poor accuracy scores and AUC only slightly higher than random guess (50%). The support vector machine baseline model’s performance is especially poor due to low-dimensionality and high collinearity among the power spectral density-based features. Figure 6b shows the ROC curves for the baseline models with visibly large steps, which appear because classifiers assign similar probability scores to many samples, providing evidence that spectral bandpower features do not strongly separate late PTS patients from patients without late PTS.

Table 4 shows the performance of the machine learning models trained on baseline features. The results show low accuracy, precision, AUC,and sensitivity scores for all models, which demonstrate the poor performance of power spectral density features in predicting late PTS.

## Discussion

Multi-fractal analysis is emerging in both neurophysiological and neuroimaging research^48, 48^. Multifractal features provide an opportunity to capture comprehensive multi-scale nuances of neuronal dynamics, reflecting the progressive changes in brain organization and the development of epileptic networks^43, 49–52^. This finding is in line with studies indicating that changes in fractality or altered multifractal spectra are associated with pathological brain states, including sleep^26^, stroke^53^ and other brain pathologies^34^. Some studies have applied mutli-fractal detrended fluctuation analysis (MFDFA) to analyze EEG signals in epilepsy patients^43, 50^ and some other pathologies including mild TBI^54^. To our knowledge, this is the first study of its kind to analyze generalized Hurst exponents computed using MFDFA from EEG data in moderate to severe TBI patients to investigate the relationship between acute post-injury brain network changes and the subsequent development of post-tramautic epilepsy. Unlike previous works that studied multi-fractal properties primarily for differentiating specific states in epilepsy patients (e.g., pre-ictal, ictal, post-ictal)^43, 50^, our research uniquely focuses on cases where the first seizure event may occur months or even years after the initial TBI and EEG recording, or may not occur at all.

The main result of our study is that patients with late PTS generally showed a larger multifractal spectrum in comparison to patients who did not experience any seizures within 2 years after the trauma, which reflects greater variability in heterogeneity of multi-scale dynamics across neural networks. These findings suggest that the specific network disruption responsible for epileptogenesis likely occurs almost immediately following the trauma. Our results are consistent with previous findings that suggest that early seizures are associated with the development of late PTS^55, 56^ and changes in fractality are evident inearly seizures^43, 57^. However, while many studies suggest that early seizures puts patients at higher risk for late PTS^55, 56, 58^, others have shown that this relationship is not always consistent^59^. In our cohort, 11 out of 33 patients with late PTS developed early PTS, and the consistent presence of multifractal changes across the larger group points to network-level alterations that extend beyond the occurrence of early seizures.

Our results align with the previous studies that showed that occurrence of certain patterns reflecting early changes in electrical brain activity are associated with PTE development in the acute period of TBI in both animal and human research^19, 60–62^. Our results provide evidence that generalized Hurst exponents can capture not only changes occurring immediately before seizures, but can also capture significant changes days to years before seizure development.

Recent neuroimaging studies highlight that damage to specific cortical regions, most notably the temporal lobe as well as frontal and parietal lobes is closely linked with the development of PTE following TBI^55, 63–65^. We observed that the CZ (central midline, roughly overlying the central sensorimotor/parietal region) and PZ (parietal midline) electrode sites consistently show the largest effect sizes when comparing late PTS and no late PTS groups (see Figure 4b). Furthermore, our results from the generalized Hurst exponents (see Figure 3a) reinforce this finding, showing clear separation at CZ and PZ, with additional but smaller effects at FZ (frontal midline) and the temporal electrodes (T3, T4, T5, T6). In our dataset, we did not observe a statistically significant correlation between contusion or ICH location and outcome after FDR correction. However, we noted a strong trend suggesting that temporal lobe ICH was associated with the occurrence of both early and late PTS.

Our study provides compelling evidence that multifractal analysis of acute EEG recordings captures pathophysiologically meaningful alterations in brain dynamics following moderate-to-severe TBI. Specifically, generalized Hurst exponents reflecting the multifractal scaling properties of the EEG demonstrate significant differences between patients who subsequently develop late PTS and those who do not (see Figure 4a). Furthermore, our classification results support the notion that generalized Hurst exponents, which quantify multifractal properties, are superior for predicting late PTS over tradition power spectral density measures (see Figure 5 and Figure 6).

Since fractality properties of EEG data are highly dependent on current brain state^66^ and age^67^, we adjusted our prediction over age and GCS score, which was specifically assessed on the first available day the EEG was recorded. It is important to mention here that we could not systematically control effects of sedation, which also highly influence the fractality present in EEG data^68^. While sedative medications such as propofol, which is broadly used in moderate to severe TBI cases, are known to decrease EEG fractal dimension^69^, it is important to note that we did not account for the exact dosage and timing of sedative agents administered to patients during EEG monitoring, as all patients were managed according to standard clinical protocols and routine clinical practice. However, the majority of studies that showed the differences in EEG features in acute TBI developing into PTE also did not account for exact dosage and timing of sedative medication given to the patientsb since they were also acting under the same assumption that all the enrolled patients were diagnosed with moderate to severe TBI and were treated according to the standard clinical protocol^19^.

Our results extend and complement prior work that has mainly focused on morphological and frequency-based features, such as power spectral density^19^, and the early appearance of epileptiform patterns on acute EEG, such as high frequency oscillations^61^. Although substantial efforts have been made to identify early biomarkers of PTE, no universally accepted clinical features have been established to date. For this reason, we did not compare the performance of our results against a gold standard, as no such standard currently exists^20, 21^. However, we do compare our results to the classical power spectral density frequency bands, where we show that generalized Hurst exponents significantly outperform to classify late PTS patients (see Figure 5 and Figure 6). Unlike power spectral density-based features, which are computed using second-order statistics, generalized Hurst exponents can capture higher-order statistical properties, including nonlinear fluctuations and long-range correlations. Hence, we observe a better performance in classifying late PTS using our model trained with generalized Hurst exponents as compared with the baseline model, which was trained with power spectral density features.

From a translational standpoint, the integration of multifractal EEG analysis into clinical workflows could substantially improve risk stratification and individualized care for TBI survivors. Routine EEG is already widely available in neurocritical care; thus, augmenting existing protocols with multifractal analysis and machine learning classification could facilitate earlier identification of high-risk patients. This could enable more intensive monitoring, timely initiation of prophylactic anti-seizure treatments, or enrollment into clinical trials for anti-epileptogenic therapies. Moreover, our approach requires only the first day of EEG recording, making it feasible for rapid, clinically actionable prognostication.

One potential limitation of this study is the relatively small sample size, especially for deep learning models, raises concerns regarding overfitting and limits generalizability. Although our exclusion criteria minimized confounding, the relatively small sample size may also restrict the applicability of our findings to broader TBI populations. Future longitudinal studies could further elucidate the temporal evolution of multifractal EEG features and their relationship to seizure onset, recurrence, and long-term outcomes. Future work will focus on integrating EEG with other modalities (advanced imaging, serum or genetic biomarkers) to yield more robust, multimodal prognostic tools. Ultimately, the development of an automated, real-time multi-fractal EEG pipeline and its incorporation into clinical decision-support systems may transform TBI care and reduce the burden of PTE.

In summary, our study demonstrates that multifractal analysis of EEG provides promising, biologically plausible biomarkers for PTE risk after severe TBI. In future, With further validation and refinement, these techniques may open new avenues for individualized monitoring, prevention, and intervention in TBI patients, a high-risk population.

## Figures and Tables

**Table 1.** TBI Patient Metadata

Variable	No Late PTS: N=33^1^	Late PTS: N=33^1^	p-value^2^	q-value^3^
**Age**			0.60	> 0.99
Median [Q1,Q3]	46[26, 58]	34[27, 53]		
Mean ± SD	44±21	40±18		
**GCS Day 0**			**0.040**	0.10
Median [Q1,Q3]	9.0[7.0, 13.0]	7.0[5.0, 9.0]		
Mean ± SD	9.2±3.7	7.2±3.4		
**Sex**			> 0.99	> 0.99
Female	6(18%)	7(21%)		
Male	27(82%)	26(79%)		
**Mechanism (collapsed)**			0.97	> 0.99
Direct impact	5(15%)	4(12%)		
Fall	2(6.1%)	2(6.1%)		
Fall > 1 m	4(12%)	5(15%)		
Gunshot wound	0(0%)	1(3.0%)		
Motor vehicle (traffic)	5(15%)	6(18%)		
Motorcycle	6(18%)	5(15%)		
Other/unspecified	5(15%)	6(18%)		
Pedestrian vs auto	6(18%)	4(12%)		
**Early PTS**			**0.004**	0.020
No Early PTS	32(97%)	22(67%)		
Early PTS	1(3.0%)	11(33%)		

1 n (%).

2 Wilcoxon rank sum test; Pearson’s Chi-squared test.

3 False discovery rate correction for multiple testing.

**Table 2.** Features used in our model and the baseline model, where all features are computed from EEG data on the first available day

Model	Ours	Baseline
Features	generalized Hurst exponents (H(q)) for q∈{−20,−19.5,…,19.5,20} Glasgow Coma Scale (GCS) Age	mean δ bandpower across channels standard deviation of δ bandpower across channels variance of δ bandpower across channels variance of θ bandpower across channels mean 95th percentile Hilbert envelope across channels std 95th percentile Hilbert envelope across channels Glasgow Coma Scale (GCS) Age

**Table 3.** Comparison of machine learning classification models trained on generalized Hurst exponents

	Accuracy(%)	AUC(%)	Precision(%)	Sensitivity(%)	Runtime(S)
Logistic Regression	63.65±3.38	72.33±3.40	59.49±3.74	64.60±2.98	**0.0013±0.0002**
SVM	89.91±3.28	94.10±3.02	88.67±3.86	89.43±4.55	0.0085±0.0020
Random Forest	**95.06±3.00**	**98.70±1.13**	**94.12±3.48**	**95.17±3.29**	0.0352±0.0003
DNN	74.99±4.76	84.16±4.88	77.14±5.34	64.37±9.09	0.1573±0.0040

**Table 4.** Comparison of machine learning models trained using power band frequencies

	Accuracy (%)	AUC (%)	Precision (%)	Sensitivity (%)	Runtime (s)
Logistic Regression	54.62±6.88	56.61±15.94	51.21±7.17	**60.00±9.13**	0.01±0.00
RBF-SVM	53.08±9.58	**61.43±9.87**	47.78±14.49	40.00±25.28	**0.00±0.00**
Random Forest	**62.31±20.42**	59.17±29.11	**53.31±24.39**	53.33±38.01	0.35±0.06
DNN	51.65±9.13	60.60±24.00	50.06±11.74	**60.00±25.28**	6.93±2.02

## Data Availability

The code is available on Github https://github.com/Undaria23/A-Multifractal-Guided-Machine-Learning-Framework-for-Late-Post-Traumatic-Seizure-Prediction. The data are available upon request at https://dabi.loni.usc.edu.
